# Effects of different doses of alfentanil on tracheal intubation stress responses in ambulatory hysteroscopic surgery: a randomized, double-blind, clinical comparative study

**DOI:** 10.3389/fmed.2026.1787000

**Published:** 2026-02-20

**Authors:** Xi Zha, Xia Ju, Jinjuan Duan, Siqi Xu

**Affiliations:** Department of Anesthesiology, The Anqing Medical Center of Anhui Medical University, Affiliated Anqing Hospital of China Pharmaceutical University, Anqing Municipal Hospital, Anqing, China

**Keywords:** adverse effects, alfentanil, hemodynamics, hysteroscopic surgery, pain, stress response

## Abstract

**Background:**

This randomized, double-blind, clinical comparative study aims to compare the tracheal intubation stress responses of different doses of alfentanil in anesthesia induction for ambulatory hysteroscopic surgery, as well as their impact on the quality of early postoperative recovery.

**Methods:**

This study enrolled a total of 160 patients aged 20–60 years scheduled for ambulatory hysteroscopic surgery. All patients underwent tracheal intubation under general anesthesia and were randomly allocated into four groups using a random number table. The low-dose alfentanil group (group AL) patients received an induction dose of 20 μg/kg alfentanil, the medium-dose alfentanil group (group AM) patients received 30 μg/kg alfentanil, the high-dose alfentanil group (group AH) received 40 μg/kg alfentanil, and the Fentanyl Group (group F) patients received an induction dose of 4 μg/kg fentanyl, with all groups additionally administered midazolam, cisatracurium, and propofol as part of the standardized induction protocol. Anesthesia maintenance was maintained with propofol and remifentanil in all groups. The primary outcome measure was the plasma norepinephrine (NE) level at 1 min after tracheal intubation (T_2_). Secondary outcomes included plasma NE and epinephrine (E) levels at baseline (T_0_, before anesthesia induction), T_2_ (excluding NE as it was the primary measure), and 5 min post-intubation (T_3_), along with mean arterial pressure (MAP) and heart rate (HR) at T_0_, the time of the insertion of the tracheal tube (T_1_), T_2_, T_3_, 5 min after the removal of the tracheal tube (T_4_); additionally, extubation time, awakening time, post-anesthesia care unit (PACU) stay duration, Steward score and numerical rating scale (NRS) pain score during the PACU stay were evaluated. Safety endpoints comprised the incidence of hypertension, hypotension, tachycardia, and bradycardia from anesthesia induction to 5 min after tracheal intubation, as well as adverse events such as nausea, vomiting, and agitation during the emergence period.

**Results:**

The levels of plasma NE and E in group AL were significantly higher than that in group F at T_2_, the levels of plasma NE and E in group AM were significantly lower than that in group AL at T_2_ ~ T_3_, and the levels of plasma NE and E in group AH were significantly lower than that in group AL and group F at T_2_ ~ T_3_ (*p* < 0.05). Compared with group F, MAP and HR in group AL were significantly higher at T_2_ (*p* < 0.05). Compared with group AL, MAP and HR in group AM were significantly lower at T_1_ ~ T_3_ (*p* < 0.05), the incidence of hypertension and tachycardia from anesthesia induction to 5 min after tracheal intubation was significantly lower in group AM (*p* < 0.05). Compared with group AL and group F, MAP and HR in group AH were significantly lower at T_1_ ~ T_3_ (*p* < 0.05), the incidence of hypotension and bradycardia was significantly higher from anesthesia induction to 5 min after tracheal intubation, and the incidence of hypertension and tachycardia was significantly lower (*p* < 0.05). The NRS pain scores at the time of extubation in group AL were significantly higher than in groups AM, AH, and F (*p* < 0.05).

**Conclusion:**

In ambulatory hysteroscopic surgery, intravenous 30 μg/kg and 40 μg/kg alfentanil inhibited the stress reaction caused by tracheal intubation, alleviated hemodynamic fluctuations, and decreased the intensity of postoperative pain. However, 40 μg/kg alfentanil significantly increased the incidence of hypotension and bradycardia from anesthesia induction to 5 min after tracheal intubation. Therefore, 30 μg/kg alfentanil may be more suitable for the application of ambulatory hysteroscopic surgery.

**Clinical trial registration:**

https://www.chictr.org.cn, ChiCTR2300073590.

## 1 Introduction

In recent years, hysteroscopy has gradually become a common method for the diagnosis and treatment of gynecological diseases. As an ambulatory surgery, it has advantages including short operation time, low bleeding, and fast recovery (1). However, intraoperative procedures such as dilation of the uterine cavity and scraping are often required, which often cause pain and discomfort to the patients, and trigger different degrees of stress response, therefore the patients’ demand for comfortable medical treatment is high (2). Total intravenous anesthesia can make patients comfortable and pain-free throughout the whole process (3). Opioids are often combined in clinical practice to strengthen the analgesic effect and reduce the risk of cardiovascular adverse events (4). However, the induction of anesthesia with opioids often causes patients to experience adverse reactions such as choking and respiratory depression, nausea and vomiting (5, 6), which affects the rapid recovery of patients after surgery, so the appropriate choice of opioids is crucial (7–10).

Alfentanil and fentanyl are both μ-opioid receptor agonists, the safety and efficacy of both have been unanimously recognized in clinical practice. Studies have shown that alfentanil and fentanyl can inhibit the stress response of tracheal intubation in general anesthesia (11). Alfentanil, as a fentanyl derivative, has a faster onset of action (1–2 min) and a shorter duration of action (about l/3 of that of fentanyl) compared with fentanyl. Its analgesic potency is about 1/5 of fentanyl, so it is more suitable for ambulatory surgery or short surgery. Xu et al. (12) found that pretreatment with low-dose alfentanil (2 μg/kg) effectively reduced both the incidence and severity of sufentanil-induced cough during the induction of anesthesia without increasing adverse events and complications. The study by Gao et al. (13) showed that 10–12 μg/kg alfentanil had less unexpected hemodynamic events, earlier anesthesia emergence, and better postoperative analgesia compared with sufentanil. At present, alfentanil has been widely used in the areas of sedation, analgesia, stress suppression, anesthesia induction, and maintenance with good results (14–16).

Ambulatory hysteroscopic surgery is the process of removing and treating abnormal lesions in the uterine cavity on the basis of examination clinically, the operation time is longer than that of examination, and the stimulation is larger. A study showed that alfentanil exhibited a reduced propensity to elicit hypoxemia and post-operative nausea and vomiting (PONV) during daytime hysteroscopy procedures. Additionally, it demonstrated a capacity for maintaining more consistent hemodynamics and better oxygen saturation compared to sufentanil (17). This finding indicates that alfentanil may be a safer option for daytime hysteroscopy surgery. According to the diagnosis and treatment norms of Anqing Municipal Hospital, all ambulatory hysteroscopic surgeries are performed under general anesthesia with endotracheal intubation. General anesthesia with tracheal intubation can effectively manage the airway safely, but the strong reaction to tracheal intubation tends to cause a sharp rise in catecholamines, as well as in blood pressure (BP) and heart rate (HR) of the patient’s body, and it may induce myocardial ischemia and arrhythmia (18, 19). Stress activates two major pathways in the body: the pituitary-adrenocortical system and the sympathetic-adrenomedullary system. Sympathetic-adrenomedullary system activation causes the release of norepinephrine (NE) from peripheral nerve terminals and epinephrine (E) from the adrenal medulla. Blood levels of these catecholamines are used as indices of sympathetic activity. Stress increases catecholamine levels in peripheratissues, blood, urine, and brain by enhancing catecholamine synthesis and by diminishing catecholamine degradation (20). Likewise, Ward et al. (21) found that E and NE increased significantly in response to the stressors. This reaction to tracheal intubation is most pronounced at 1 min after tracheal intubation and gradually decreases at 5 min after tracheal intubation (22, 23). Therefore, in this study, blood samples were collected from patients at 1 and 5 min after tracheal intubation for testing the levels of plasma NE and E. Since the analgesic potency ratio of fentanyl to alfentanil is 5:1, 4 μg/kg fentanyl group was chosen to compare with the 20 μg/kg alfentanil group in this study.

At present, the clinical effects of different doses of alfentanil in the application of ambulatory hysteroscopic surgery have not been reported. This study aims to compare tracheal intubation stress responses of different doses of alfentanil combined with propofol for the induction of anesthesia in ambulatory hysteroscopic surgery, as well as their impact on early postoperative recovery quality. The goal is to identify a more precise and comfortable anesthetic regimen for ambulatory hysteroscopic surgery.

## 2 Materials and methods

### 2.1 Study design and ethical statements

This randomized, double-blind, clinical comparative study was reviewed and approved by the Ethics Committee of the Anqing Medical Center of Anhui Medical University (Medical Ethics Approval No. 2023Z1003) and prospectively registered in the Chinese Clinical Trial Registry (ChiCTR2300073590; principal investigator: Xi Zha; registration date: July 14, 2023). The entire process of this study was conducted at Anqing Municipal Hospital, Anqing City, Anhui Province, China, and was divided into four consecutive stages with clear time nodes and specific tasks: the first stage was the ethical review of the hospital, which was approved on June 21, 2023; the second stage was the pre-registration at the Chinese Clinical Trial Registry, completed on July 14, 2023; the third stage was case collection, carried out from January 2024 to August 2024; and the fourth stage included data statistics and paper writing, conducted from September 2024 to June 2025. All participants provided their written informed consent to participate in this study. All procedures of this trial followed the tenets of the Helsinki Declaration.

### 2.2 Participants and recruitment

This prospective trial enrolled patients aged 20 to 60 years with a body mass index (BMI) of 18–28 kg/m^2^ and American Society of Anesthesiologists (ASA) class I or II who underwent ambulatory hysteroscopic surgery under general anesthesia with written informed consent obtained under Institutional Review Board supervision. The exclusion criteria were severe depression or long-term use of psychiatric medications, a history of mental illness, patients with severe heart, lung, brain, liver, kidney or endocrine diseases, long-term use of sedative or analgesic medications; preoperative pulmonary infections or respiratory failure; allergic to opioids and other anesthetic drugs.

### 2.3 Randomization and blinding

160 female patients who underwent ambulatory hysteroscopic surgery under general anesthesia with tracheal intubation from January to August 2024 were randomized into 4 groups according to 1:1:1:1 using computer generated random numbers: 20 μg/kg alfentanil group (group AL), 30 μg/kg alfentanil group (group AM), 40 μg/kg alfentanil group (group AH) and 4 μg/kg fentanyl group (group F). All drugs used in this study, including fentanyl and alfentanil, were purchased through official and legitimate channels from the hospital pharmacy. An opaque envelope was used to hide the grouping information, and a nurse anesthetist configured the medications according to the groupings, other personnel (including postoperative followers, preoperative followers, surgeons, surgical nurses, patients, and anesthesiologists) were unaware of the grouping.

### 2.4 Anesthesia methods

All patients were fasted for 8 h and dehydrated for 4 h preoperatively. After admission to the operation room, intravenous access was opened, electrocardiogram (ECG), saturation of pulse oxygen (SpO_2_), mean arterial pressure (MAP), and HR were routinely monitored (Philips, Germany), mask preoxygenation (oxygen concentration 100%) was given at an oxygen flow rate of 6–8 L/min, 10 mg dexamethasone (Hainan Bete Pharmaceutical Co., Ltd. China; NMPA Approval No: H32021561) and 0.5 mg penehyclidine (Chongqing Huasen Pharmaceutical Co., Ltd. China; NMPA Approval No: H20193271) were given 5 min before the induction of anesthesia in all groups. After 5 min of oxygen denitrification, 0.01 mg/kg midazolam (Jiangsu Nhwa Pharmaceutical Co., Ltd., China; NMPA Approval No. H10980025), 0.1 mg/kg cisatracurium (Zhejiang Xianju Pharmaceutical Co., Ltd. China; NMPA Approval No. H20090198), and 2 mg/kg propofol (Xi’an Lipan Pharmaceutical Company, China; NMPA Approval No. H19990282) were injected in all groups. In the fentanyl (Yichang Humanwell Pharmaceutical Co., Ltd. China; NMPA Approval No. H20003688) group, 4 μg/kg fentanyl was injected intravenously for anesthesia induction, the injection was completed in 30 s. In the alfentanil (Jiangsu Nhwa Pharmaceutical Co., Ltd., China; NMPA Approval No. H20213853) groups, 20 μg/kg, 30 μg/kg, and 40 μg/kg alfentanil were given in groups AL, AM, and AH, respectively for anesthesia induction, the injection was completed in 30s. When the patient’s consciousness disappeared and the jaw relaxed, the tracheal tube was inserted. After successful insertion of the tracheal tube, the anesthesia machine (Dräger Fabius® Plus, Germany) was connected, volume-controlled ventilation (VCV) mode was used for mechanical ventilation, the respiratory parameters were adjusted as follows: tidal volume (VT) 6–8 mL/kg, respiratory rate (RR) 10–12 beats per min (bpm), inspiratory/expiratory ratio (I:E) was set at 1:2, end-tidal carbon dioxide partial pressure (P_et_CO_2_) was maintained at a level of between 35 and 45 mmHg, and perioperative inspired oxygen concentration was set at 40–50%. Intraoperative anesthesia was maintained with propofol (Xi’an Lipan Pharmaceutical Company, China; NMPA Approval No. H20123318) 4 mg/kg/h and remifentanil (Yichang Humanwell Pharmaceutical Co., Ltd., China; NMPA Approval No. H20030197) 0.1 μg/kg/min.

During the induction of anesthesia and surgical procedure, anesthesia was appropriately deepened in case of tachycardia (HR > 100 bpm) and hypertension (MAP ≥100 mmHg), atropine (0.3 mg) was intravenously given in case of bradycardia (HR < 50 bpm), ephedrine (6 mg) was intravenously given in case of hypotension (MAP ≤65 mmHg) (24). Intraoperative fluctuations in MAP and HR were maintained at 20% of basal values. The patient was transferred to the post-anesthesia care unit (PACU) with an endotracheal tube at the end of the operation. When the patient was fully awakened, the endotracheal tube was removed. The Steward score is a widely clinical assessment scale used to evaluate the recovery status of patients after general anesthesia, especially in the PACU, which quantifies the degree of recovery through three core dimensions: consciousness, airway patency, and motor responsiveness, with a total score ranging from 0 to 6 points. Specifically, the scoring criteria are as follows: 1. Consciousness: 0 point indicated no response to stimulation; 1 point indicated drowsiness with response to stimulation but confusion; 2 point indicated fully awake; 2. Airway: 0 point indicated requiring assistance to maintain airway patency; 1 point indicated maintaining good airway patency; 2 point indicated actively crying or coughing on command; 3. Movement: 0 point indicated no voluntary moving; 1 point indicated moving limbs weakly; 2 point indicated moving limbs purposefully (25). A total Steward score of 4 or higher is the general standard for a patient to meet the discharge criteria from the PACU. In this study, the patient was sent back to the ward when the Steward score was ≥5.

### 2.5 Measurements

2 mL of venous blood was drawn from patients before induction of anesthesia (T_0_), 1 min after tracheal tube insertion (T_2_), and 5 min after tracheal tube insertion (T_3_) respectively, and stored at −20 °C. After centrifugation, the supernatant was taken, and the plasma NE and E level was determined by high-performance liquid chromatography tandem mass spectrometry.

#### 2.5.1 Primary observational outcome was the levels of plasma NE at T_2_

Secondary observational indexes: (1) the levels of plasma NE at T_0_ and T_3_, the levels of plasma E at T_0_, T_2_, T_3_; (2) MAP and HR were recorded before anesthesia induction (T_0_), at the time of tracheal tube insertion (T_1_), 1 min after tracheal tube insertion (T_2_), 5 min after tracheal tube insertion (T_3_), and 5 min after removal of the tracheal tube (T_4_); the occurrence of hypertension, hypotension, tachycardia, and bradycardia was recorded from anesthesia induction to 5 min after tracheal intubation; (3) the Sedation-Agitation Scale (SAS) was used to assess the patients’ agitation during awakening, the SAS score totaled 7 points: 1 point indicated inability to awaken; 2 points indicated very sedated; 3 points indicated sedated and drowsy; 4 points indicated quiet and cooperative; 5 points indicated agitated; 6 points indicated very agitated; and 7 points indicated dangerously agitated (26), a score of ≥5 on the SAS score was regarded as the occurrence of awakening-phase agitation. Numerical rating scale (NRS) pain scores were used to assess the pain of patients at the moment of extubation, 10 min after extubation, and when leaving the PACU, The total NRS pain scores were 10 points: 0 points: no pain; 1–3 points: mild pain; 4–6 points: moderate pain; 7–10 points: severe pain (27), flurbiprofen axetil 50 mg was injected intravenously at a score of ≥4. The occurrence of nausea and vomiting during the stay in the PACU was recorded, and if nausea greater than or equal to 2 times and vomiting occurred, it should be handled with ondansetron 8 mg intravenously. (4) The extubation time (the time from the discontinuation of anesthesia drugs to the extubation of the tracheal tube), awakening time (the time from the discontinuation of anesthesia drugs to the time the patient was able to act according to the instructions), duration of PACU stay, and Steward scores were recorded.

Baseline characteristics recorded for patients included age, BMI, ASA classification, ethnic categorization, the proportion of smokers and drinkers, comorbidities, educational attainment, duration of surgery (the duration from the start of the surgery to its completion), duration of anesthesia (the time from the administration of anesthesia drugs to the end of anesthesia), dose of maintenance anesthetic agents, and types and doses of vasoactive drugs administered intraoperatively.

### 2.6 Statistical analysis

The sample size calculation was performed using PASS 15.0 software based on preliminary pre-experimental study results. The levels of plasma NE at T_2_ of the subjects were used as the main evaluation index, and the average value in Group F was 554.5 ± 52.30. Assuming *α* = 0.05 and *β* = 0.2, 32 cases were needed in each group, 40 cases were estimated to be included in each group accounting for the 20% failure rate.

SPSS V27.0 software was used for statistical analysis. Normally distributed measurement data were expressed as mean ± standard deviation (SD, 𝑥̄ ± *s*), within-group comparisons were made by repeated measures analysis of variance (ANOVA), between-group comparisons were made by one-way ANOVA, and Bonferroni correction was applied. Data that did not conform to normal distribution were expressed as median [interquartile range (IQR)], and analyzed by the Kruskal–Wallis test; count data were expressed as the number of cases and the *n*-percentage (%), and between-group comparisons were made by the *χ*^2^ test or the Fisher’s exact test. The *p* value <0.05 was considered statistically significant.

## 3 Results

**Figure 1:**
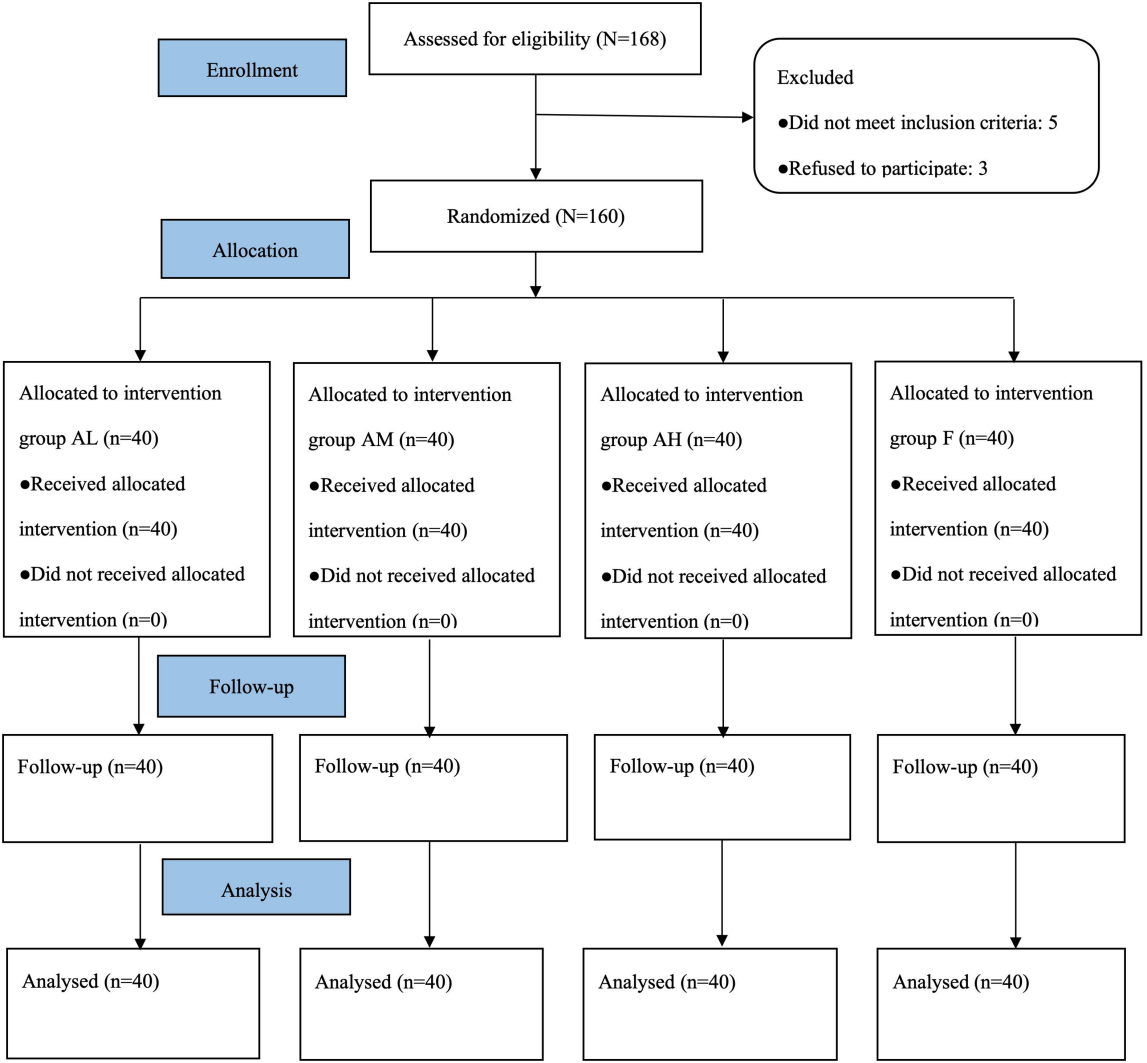
Study flow diagram. A total of 168 patients were initially included in the study, of whom 5 were excluded for failure to meet inclusion criteria and 3 declined participation. Ultimately, 160 patients (*N* = 160, 40 in each group) completed the trial.

A total of 168 patients were recruited in this study (*N* = 168), 5 patients did not meet the inclusion criteria and 3 patients refused to participate, 160 cases were finally enrolled (*N* = 160), 40 cases in each group, and all successfully completed the trial (Figure 1). A comprehensive analysis of baseline characteristics revealed no statistically significant intergroup disparities (*p* > 0.05) (Table 1).

**Table 1:** Baseline data of all patients.

Variables	Group AL (*n* = 40)	Group AM (*n* = 40)	Group AH (*n* = 40)	Group F (*n* = 40)	*p*-value
Age (years)	42.0 ± 5.5	46.3 ± 8.4	42.1 ± 8.2	39.0 ± 7.4	0.206
BMI (kg/m^2^)	23.9 ± 1.9	22.0 ± 2.5	23.2 ± 2.2	22.0 ± 2.6	0.190
ASA (I/II)	8/32	10/30	8/32	14/26	0.395
Smoker, *n* (%)	4 (10)	3 (7.5)	4 (10)	6 (15)	0.741
Drinker, *n* (%)	5 (12.5)	6 (15)	5 (12.5)	7 (17.5)	0.906
Ethnic categorization					
The Han people	37 (92.5)	38 (95)	39 (97.5)	37 (92.5)	0.730
Chinese minority	2 (5)	1 (2.5)	1 (2.5)	3 (7.5)	0.650
Others	1 (2.5)	1 (2.5)	0 (0)	0 (0)	0.567
Level of education					
Primary school, *n* (%)	8 (20)	9 (22.5)	7 (17.5)	9 (22.5)	0.936
Middle school, *n* (%)	12 (30)	9 (22.5)	11 (27.5)	13 (32.5)	0.781
High school, *n* (%)	11 (27.5)	10 (25)	12 (30)	8 (20)	0.766
College or higher, *n* (%)	9 (22.5)	12 (30)	10 (25)	10 (25)	0.891
Comorbidity					
Hypertension, *n* (%)	5 (12.5)	2 (5)	3 (7.5)	4 (10)	0.667
Coronary heart disease, *n* (%)	0 (0)	2 (5)	1 (2.5)	0 (0)	0.291
Diabetes, *n* (%)	2 (5)	4 (10)	4 (10)	3 (7.5)	0.135
Anaemia, *n* (%)	19 (47.5)	20 (50)	22 (55)	24 (60)	0.687
Operation time (min)	20.5 ± 7.6	19.4 ± 9.0	24.2 ± 10.0	20.0 ± 5.3	0.556
Anesthesia time (min)	29.1 ± 9.2	25.7 ± 9.0	32.6 ± 10.6	28.0 ± 7.5	0.410
Propofol dose (maintenance)	135 ± 44.8	124.2 ± 27.6	131 ± 45.0	120.6 ± 32.8	0.274
Remifentanil dose	0.223 ± 0.12	0.185 ± 0.04	0.271 ± 0.12	0.208 ± 0.05	0.128
The number of patients using ephedrine, *n* (%)	6 (15)	10 (25)	14 (35)	8 (20)	0.185
The dose of ephedrine (mg)	6 (6, 9.75)	6 (6, 9)	10.5 (6, 12)	7.5 (6, 9)	0.333
The number of patients using atropine, *n* (%)	7 (17.5)	9 (22.5)	15 (37.5)	8 (20)	0.154
The dose of atropine (mg)	0.3 (0.3, 0.35)	0.3 (0.3, 0.5)	0.5 (0.3, 0.5)	0.3 (0.3, 0.3)	0.265

Data are presented as mean ± SD, median (interquartile range), or number (percentage).

Group AL, 20 μg/kg alfentanil group, Group AM; 30 μg/kg alfentanil group; Group AH, 40 μg/kg alfentanil group; Group F, 4 μg/kg fentanyl group; BMI, body mass index; ASA, American Society of Anesthesiologists; operation time, the duration from the start of the surgery to its completion; anesthesia time, the time from the administration of anesthesia drugs to the end of anesthesia.

### 3.1 NE and E levels in plasma at different time points

There were no significant differences in the pre-induction NE and E levels among the four groups. Compared with T_0_, the levels of plasma NE and E in group AL were significantly higher at T_2_ (*p* < 0.001, *p* < 0.001), the levels of plasma NE and E in group AM were significantly lower at T_3_ (*p* < 0.001, *p* < 0.001), and in group AH were significantly lower at T_2_ ~ T_3_ (all *p* < 0.001). The levels of plasma NE and E in group AH were significantly lower than groups AL and F at T_2_ ~ T_3_ (*p* < 0.001, *p* = 0.018, *p* = 0.001, *p* = 0.006, *p* < 0.001, *p* = 0.001, *p* = 0.005, *p* = 0.011). Compared with group AL, the levels of plasma NE and E in group AM were significantly lower at T_2_ ~ T_3_ (*p* = 0.007, *p* = 0.001, *p* = 0.005, *p* < 0.001). The levels of plasma NE and E in group AL were higher than that in group F at T_2_ (*p* = 0.003, *p* = 0.001). There were no significant differences in plasma NE and E levels between group AM and group F at T_2_ ~ T_3_ (Table 2).

**Table 2:** Comparison of plasma NE and E levels at different time points.

Variables		Group AL (*n* = 40)	Group AM (*n* = 40)	Group AH (*n* = 40)	Group F (*n* = 40)	*p*-value
NE (pmol/L)	T_0_	562.58 ± 53.21	566.68 ± 53.09	551.75 ± 62.21	557.0 ± 63.81	0.862
	T_2_	605.15 ± 56.27^ac^	563.05 ± 50.61^b^	510.65 ± 57.38^abc^	567.5 ± 54.60	0.005
	T_3_	556.75 ± 50.81	522.84 ± 50.12^ab^	475.75 ± 55.39^abc^	541.5 ± 45.84	0.006
E (pmol/L)	T_0_	174.8 ± 47.3	176.0 ± 42.1	165.1 ± 50.8	171.4 ± 45.4	0.142
	T_2_	220.3 ± 48.8^ac^	149.3 ± 39.8^b^	140.8 ± 47.8^abc^	178.7 ± 52.3	0.002
	T_3_	169.7 ± 48.5	132.6 ± 40.3^ab^	126.4 ± 47.6^abc^	160.6 ± 46.2	0.001

Data are presented as mean ± SD. Compared with T_0_, ^a^*p* < 0.05; compared with group AL, ^b^*p* < 0.05; compared with group F, ^c^*p* < 0.05.

NE, norepinephrine; E, epinephrine; T_0_, before anesthesia induction; T_2_, 1 min after tracheal tube insertion; T_3_, 5 min after tracheal tube insertion.

### 3.2 MAP and HR at different time points

There were no significant differences in MAP and HR before induction and 5 min after extubation among the four groups. Compared with T_0_, patients in group AL had significantly higher MAP and HR at T_1_ ~ T_2_ (*p* < 0.001, *p* = 0.004, *p* = 0.001, *p* = 0.008); patients in group AM had significantly lower MAP and HR at T_2_ ~ T_3_ (*p* = 0.022, *p* = 0.002, *p* = 0.032, *p* = 0.002), patients in group AH had significantly lower MAP and HR at T_1_ ~ T_3_ (all *p* < 0.001); patients in group F had significantly lower MAP and HR at T_3_ (*p* = 0.002, *p* = 0.006). Compared with groups AL and F, patients in group AH had significantly lower MAP and HR at T_1_ ~ T_3_ (*p* < 0.001, *p* = 0.001, *p* < 0.001, *p* < 0.001, *p* < 0.001, *p* < 0.001, *p* = 0.001, *p* = 0.004, *p* < 0.001, *p* = 0.015, *p* = 0.002, *p* = 0.001). Compared with group AL, patients in group AM had significantly lower MAP and HR at T_1_ ~ T_3_ (*p* = 0.002, *p* < 0.001, *p* = 0.012, *p* = 0.002, *p* = 0.005, *p* = 0.001). MAP and HR were significantly higher in group AL compared with group F at T_2_ (*p* = 0.025, *p* = 0.005). There were no significant differences in MAP and HR between group AM and group F at T_1_ ~ T_3_ (Table 3).

**Table 3:** Comparison of MAP and HR at different time points.

Variables		Group AL (*n* = 40)	Group AM (*n* = 40)	Group AH (*n* = 40)	Group F (*n* = 40)	*p*-value
MAP (mmHg)	T_0_	92.7 ± 9.6	92.1 ± 9.1	91.1 ± 6.5	92.1 ± 5.4	0.975
	T_1_	103.7 ± 8.7^a^	90.1 ± 9.4^b^	80.2 ± 9.0^abc^	97.2 ± 9.1	<0.001
	T_2_	101.0 ± 6.4^ac^	83.1 ± 6.1^ab^	70.1 ± 6.2^abc^	91.3 ± 9.5	<0.001
	T_3_	88.7 ± 7.9	80.9 ± 7.3^ab^	63.0 ± 6.9^abc^	83.2 ± 7.6^a^	<0.001
	T_4_	91.0 ± 8.8	91.9 ± 9.1	91.8 ± 7.7	92.5 ± 8.0	0.984
HR (bpm)	T_0_	73.8 ± 7.4	75.4 ± 8.5	74.6 ± 12.0	75.2 ± 9.8	0.763
	T_1_	85.5 ± 9.4^a^	74.3 ± 10.9^b^	65.4 ± 9.2^abc^	79.4 ± 9.4	0.006
	T_2_	82.5 ± 10.0^ac^	67.5 ± 10.2^ab^	62.3 ± 8.2^abc^	73.4 ± 6.5	<0.001
	T_3_	72.1 ± 10.7	62.8 ± 10.1^ab^	57.7 ± 7.3^abc^	66.2 ± 5.8^a^	0.004
	T_4_	73.9 ± 9.4	75.6 ± 10.1	77.2 ± 10.4	73.2 ± 10.1	0.809

Data are presented as mean ± SD. Compared with T_0_, ^a^*p* < 0.05; compared with group AL, ^b^*p* < 0.05; compared with group F, ^c^*p* < 0.05.

MAP, mean arterial pressure; HR, heart rate; T_0_, before anesthesia induction; T_1_, at the time of tracheal tube insertion; T_2_, 1 min after tracheal tube insertion; T_3_, 5 min after tracheal tube insertion; T_4_, 5 min after removal of the tracheal tube.

### 3.3 Adverse cardiovascular events from anesthesia induction to 5 min after tracheal intubation

Compared with groups AL and F, the incidence of hypotension and bradycardia from anesthesia induction to 5 min after tracheal intubation was significantly higher in group AH (all *p* < 0.001), the incidence of hypertension and tachycardia was significantly lower in group AH (*p* < 0.001, *p* = 0.003, *p* < 0.001, *p* = 0.006); the incidence of hypertension and tachycardia was significantly lower in group AM compared with group AL (*p* = 0.018, *p* = 0.022). There were no statistically significant differences in the incidence of adverse reactions between groups AL and F, and between groups AM and F (Table 4).

**Table 4:** Comparison of the incidence of adverse cardiovascular events from anesthesia induction to 5 min after tracheal intubation.

Variables	Group AL (*n* = 40)	Group AM (*n* = 40)	Group AH (*n* = 40)	Group F (*n* = 40)	*p*-value
Hypotension	4 (10)	7 (18)	14 (35)^bc^	5(13)	<0.001
Hypertension	13 (33)	5(13)^b^	2 (5)^bc^	8(20)	<0.001
Bradycardia	4 (10)	8 (20)	15(38)^bc^	7(18)	<0.001
Tachycardia	14 (35)	6 (15)^b^	2 (5)^bc^	9(23)	<0.001

Data are presented as number of patients (%). Compared with group AL, ^b^*p* < 0.05; compared with group F, ^c^*p* < 0.05.

Tachycardia, HR >100 beats per minute; hypertension, MAP ≥100 mmHg; bradycardia, HR <50 beats per minute; hypotension, MAP ≤65 mmHg.

### 3.4 Awakening time, extubation time, adverse effects during PACU stay, NRS pain scores at different time points, duration of PACU stay, Steward scores

The NRS pain scores at extubation in group AL were significantly higher than that in groups AM, AH, and F (*p* < 0.001, *p* < 0.001, *p* < 0.001), and the pain was relieved after symptomatic treatment when the NRS pain score ≥4. There were no significant differences between four groups in NRS pain scores at 10 min after extubation and leaving the PACU. There were no significant differences in awakening time, extubation time, the incidence of agitation, nausea and vomiting during the PACU stay, duration of PACU stay and Steward scores (Table 5).

**Table 5:** Comparison of awakening time, extubation time, incidence of agitation, nausea and vomiting during PACU stay, and NRS pain scores at different time points.

Variables	Group AL (*n* = 40)	Group AM (*n* = 40)	Group AH (*n* = 40)	Group F (*n* = 40)	*p*-value
Awakening time	12.7 ± 8.2	15.1 ± 6.9	16.0 ± 4.8	15.5 ± 8.4	0.415
Extubation time	13.9 ± 8.3	16.3 ± 6.8	16.9 ± 5.5	16.1 ± 9.5	0.488
Duration of PACU stay	35.9 ± 4.1	37.3 ± 4.7	36.3 ± 3.7	36.2 ± 31	0.563
Steward scores	5.4 ± 0.7	5.6 ± 0.5	5.6 ± 0.5	5.5 ± 0.5	0.946
Nausea and vomiting	0 (0)	1 (3)	0 (0)	1 (3)	1.000
Agitation	3 (8)	0 (0)	0 (0)	1 (3)	0.194
NRS scores					
Extubation	3 (3, 4)	2 (1.75, 2.25)^b^	2 (1.75, 2)^b^	2 (1.75, 2)^b^	<0.001
5 min after extubation	2 (1, 3)	2 (1.75, 2.25)	1 (1, 2)	1 (1, 2)	0.067
Leaving the PACU	1 (1, 2)	1 (1, 2)	1 (1, 2)	1 (1, 2)	0.934

Data are presented as mean ± SD, median (interquartile range), or number (percentage). Compared with group AL, ^b^*p* < 0.05.

Awakening time, the time from the discontinuation of anesthesia drugs to the time the patient was able to act according to the instructions; extubation time, the time from the discontinuation of anesthesia drugs to the extubation of the tracheal tube; Agitation, SAS score of ≥5; PACU, post-anesthesia care unit; NRS, numerical rating scale.

## 4 Discussion

The results of this study showed that 30 μg/kg and 40 μg/kg alfentanil can suppress intubation-induced stress response and reduce cardiovascular responses and postoperative pain in patients undergoing ambulatory hysteroscopic surgery, but 40 μg/kg alfentanil significantly increased the incidence of hypotension and bradycardia from anesthesia induction to 5 min after tracheal intubation.

The results showed that the levels of plasma NE, E, MAP, and HR of the patients in group AL within 1 min after intubation were significantly higher than the baseline values, it could be attributed to the immediate release of catecholamines due to sympathetic excitation, suggesting that 20 μg/kg alfentanil was not able to inhibit the stress response induced by tracheal intubation effectively. However, Li et al. (5) found that 20 μg/kg alfentanil in combination with propofol induction for general anesthesia intubation for ambulatory surgery reduced choking and smoother intraoperative hemodynamics. The study by Chen et al. (28) also found that alfentanil 20 μg/kg for rapid sequence intubation in elderly patients can effectively inhibit the violent cardiovascular reaction caused by intubation, and avoid the inhibition of cardiovascular system caused by large dose, hemodynamics more stable. The reason for the different conclusions might be related to the different study populations and propofol induction dose in this trial. In this study, with the increase in the dose of alfentanil, the MAP, HR, NE, and E at 1 min and 5 min after intubation in the 30 μg/kg and 40 μg/kg alfentanil groups were significantly lower than the baseline values, with the 40 μg/kg alfentanil group showing the most significant decrease, and the incidence of bradycardia and hypotension was significantly higher. This may be due to the significant negative correlation between the alfentanil dose and the release of E, NE, and vasopressin after tracheal intubation, as well as the elevation of arterial blood pressure (ABP) and HR (23). Some studies also found that alfentanil dilates μ receptors, reduces peripheral resistance and systemic tension, causes a decrease in BP, activates the vagus nerve, and leads to a reduction in HR (29–31). Moreover, alfentanil can enhance the inhibitory effect of propofol on systolic blood pressure (SBP) and HR (32). Furthermore, the speed of drug infusion can also affect changes in BP and HR. Therefore, the increased incidence of cardiovascular adverse reactions in the 40 μg/kg alfentanil group may be related to both the alfentanil dose and the infusion speed. In contrast, patients in the 30 μg/kg alfentanil group had the smallest fluctuation of plasma NE and E levels, MAP, and HR within 5 min after intubation, and the incidence of hypertension and tachycardia was significantly decreased, which indicated that 30 μg/kg alfentanil not only effectively suppressed the tracheal intubation response, but also resulted in more stable hemodynamics during the operation. Furthermore, no significant differences were observed between group AM and group F in NE, E, MAP, and HR at each observation time point, indicating that 30 μg/kg alfentanil is non-inferior to 4 μg/kg fentanyl in suppressing the stress response to tracheal intubation. Abou-Arab et al. (23) found that 55 μg/kg alfentanil in combination with thiopental and rocuronium for tracheal intubation was necessary to prevent BP increase beyond 10% of baseline after intubation. This is inconsistent with the findings of this study, and may be related to the different general anesthesia induction drug types, doses, and intubation time. Compared with group F, group AL had significantly higher plasma NE and E levels, MAP, and HR at 1 min after intubation, indicating that 4 μg/kg fentanyl was used for less stress response and smoother hemodynamics within 5 min after intubation compared with 20 μg/kg alfentanil, it may be related to the fact that 4 μg/kg fentanyl better suppressed tracheal intubation stress response. Regarding the issue that ephedrine can stimulate the release of endogenous NE and E, and the use of ephedrine and atropine during the operation to manage hypotension or tachycardia may cause confusion in MAP, HR and plasma NE or E levels. In this research, Table 1 revealed no statistically significant differences in the types or doses of vasoactive drugs used among the four groups. Therefore, the intraoperative administration of ephedrine and atropine did not significantly compromise the accuracy of the conclusions in this study.

Propofol has a short half-life with no accumulation effect and exerts a synergistic analgesic effect by inhibiting the cAMP signaling pathway in combination with alfentanil (33, 34). The results of this study showed that patients in group AL had significantly higher NRS pain scores than the other three groups at the time of extubation and required the highest number of analgesic remediation cases, which may be dose-dependently related to the duration and effect of analgesia of alfentanil. Despite the low incidence of hyperalgesia induced by alfentanil (35), it has a slow distribution half-life of only 14 min, the 20 μg/kg alfentanil group had a rapid decrease in drug concentration in the body due to the low dose, and the patients woke up with virtually no analgesic residual effect, resulting in a high NRS pain score. The NRS pain scores at extubation of the 4 μg/kg fentanyl group were significantly lower than that of the 20 μg/kg alfentanil group, which was probably related to the accumulation of fentanyl in the body and slow metabolism (14). However, Türk et al. (36) demonstrated in a study that 1 μg/kg fentanyl had a better sedative, analgesic effect, and shorter recovery time compared to 10 μg/kg alfentanil. Inconsistent results may be related to the dose of alfentanil or fentanyl, the type of surgery, and the fact that the surgery was not tracheal intubated.

Fast postoperative awakening with a low incidence of adverse effects promotes rapid recovery. Although there were no significant differences in the time of awakening and extubation among the four groups in this study, the time of awakening and extubation was prolonged in the fentanyl group compared with the other three groups, which may be related to the absence of opioid-like activity of the metabolites of alfentanil (15), and to the longer action duration of fentanyl than that of alfentanil (37, 38).

Arousal agitation is one of the indicators of the quality of arousal, the Sedation-Agitation Scale is a valid tool for evaluating the quality of postoperative recovery. Postoperative pain is the main cause of postoperative agitation (39), there were no significant differences in the incidence of awakening agitation among the four groups in this study, it may be related to the mild pain after hysteroscopic surgery and the good sedative effect of both alfentanil and fentanyl (16, 40). Besides, the sedative effect of remifentanil has a certain preventive effect on awakening agitation to a certain extent (41).

PONV is the most common postoperative complication of general anesthesia, which not only affects the quality of recovery but may also reduce patient postoperative satisfaction (42, 43). Zhu et al. (44) found that alfentanil did not reduce the incidence of nausea and vomiting in gynecologic ambulatory surgery. In this study, there were no statistically significant differences in the incidence of postoperative nausea and vomiting among the four groups, which may be related to the prophylactic use of dexamethasone prior to induction of anesthesia (45).

In this study, the recovery indicators including the duration of PACU stay and the Steward score were observed. There were no significant differences in these two indicators among the four groups. This suggests that in this study, the levels of stress hormones during the induction period and hemodynamic parameters are more important indicators for observing the application of alfentanil in ambulatory hysteroscopic surgery.

This study has some limitations. Firstly, since ephedrine can stimulate the release of endogenous NE and E, the use of ephedrine and atropine during the operation to manage hypotension or tachycardia may cause confusion in MAP, HR and plasma NE/E levels, which to some extent may affect the observation results. Future research should establish more time points and eliminate confounding factors to ensure the accuracy of results. Secondly, the observation period for postoperative adverse reactions such as nausea and vomiting in this study was relatively short, adverse reactions should be followed up for better clarity on the long-term effects of alfentanil. Thirdly, the analgesic drug used for anesthesia maintenance in all four groups is remifentanil, which may interfere with the experimental results of this study. Maintenance drug administration should be individualized according to the specific opioid employed in order to minimize perioperative medication errors. Fourthly, opioids exert inherent sedative properties. In the present investigation, the incidence of postoperative drowsiness was not assessed, thereby precluding direct evaluation of alfentanil-related sedative effects. Fifthly, propofol and remifentanil were administered via conventional infusion rather than target-controlled infusion during surgery. Implementation of more precise target-controlled infusion modalities throughout the procedure may have yielded more definitive and robust findings. Sixthly, due to the surgical nature of this study, all subjects were female patients, which may lead to gender bias. Future studies could compare the effects of alfentanil in surgeries applicable to both males and females. Finally, the present study was designed as a single-center investigation, which represents an inherent limitation; consequently, the results may not be fully generalizable to a broader clinical population. A sample size of 40 patients per group was adopted for analysis, yet this sample scale may be regarded as relatively modest for a clinical trial. Accordingly, future investigations with larger sample sizes, combined with multicenter, randomized, double-blind controlled studies, are warranted to further elucidate the effects of alfentanil on anesthesia induction and postoperative recovery in patients.

## 5 Conclusion

Intravenous 30 μg/kg, 40 μg/kg alfentanil can inhibit the stress response caused by tracheal intubation, reduce hemodynamic fluctuations, decrease the intensity of postoperative pain in patients undergoing ambulatory hysteroscopic surgery, but 40 μg/kg alfentanil significantly increased the incidence of hypotension and bradycardia from anesthesia induction to 5 min after tracheal intubation. Therefore, 30 μg/kg alfentanil may be more suitable for the application of ambulatory hysteroscopic surgery, indicating that it is worthy of further promotion and application in ambulatory surgeries. However, future studies with larger sample sizes and multicenter designs are warranted to further investigate the optimal dosage of alfentanil in surgeries performed under general anesthesia with endotracheal intubation.

## Data Availability

The raw data supporting the conclusions of this article will be made available by the authors, without undue reservation.

## Glossary

NE: norepinephrine
E: epinephrine
MAP: mean arterial pressure
HR: heart rate
BP: blood pressure
SBP: systolic blood pressure
PACU: post-anesthesia care unit
NRS: numerical rating scale
BMI: body mass index
ASA: American Society of Anesthesiologists
ECG: electrocardiogram
SpO_2_: saturation of pulse oxygen
VCV: volume-controlled ventilation
VT: tidal volume
RR: respiratory rate
I:E: inspiratory/expiratory ratio
P_et_CO_2_: end-tidal carbon dioxide partial pressure
SAS: Sedation-Agitation Scale
SD: standard deviation
ANOVA: analysis of variance
IQR: interquartile range
PONV: post-operative nausea and vomiting
ABP: arterial blood pressure
